# Novel SOAT inhibitors block DHEAS transport and suppress proliferation in MCF-7 breast cancer cells

**DOI:** 10.1038/s41598-026-47803-0

**Published:** 2026-04-10

**Authors:** Emre Karakus, Silke Leiting, Michael Daude, Wibke E. Diederich, Joachim Geyer

**Affiliations:** 1 Institute of Pharmacology and Toxicology, Faculty of Veterinary Medicine, Biomedical Research Center Seltersberg (BFS), Justus Liebig University of Giessen, Schubertstr. 81, 35392 Giessen, Germany; 2 Fachbereich Pharmazie, Institut für Pharmazeutische Chemie und Zentrum für Tumor und Immunbiologie, Philipps-Universität Marburg, Hans-Meerwein-Str. 3, 35043 Marburg, Germany

**Keywords:** SOAT (SLC10A6), DHEAS transport, Intracrine estrogen formation, MCF-7 cells, Breast cancer, Endocrine therapy, Biochemistry, Cancer, Cell biology, Drug discovery

## Abstract

Intracrine estrogen formation from adrenal precursors such as dehydroepiandrosterone sulfate (DHEAS) plays a critical role in sustaining estrogen receptor (ER)-positive breast cancer cell proliferation, particularly in postmenopausal women. The sodium-dependent organic anion transporter (SOAT, gene symbol *SLC10A6*) facilitates the cellular uptake of sulfated steroids, thereby making these precursors available for local estrogen biosynthesis. However, the impact of SOAT inhibition on intracrine estrogen metabolism in breast cancer remains unclear. We investigated the effects of pharmacological SOAT inhibition on SOAT-mediated DHEAS transport, cell proliferation and intracrine estradiol synthesis in SOAT-overexpressing MCF-7 breast cancer cells (MCF-7_SOAT). These MCF-7_SOAT cells were treated with the SOAT inhibitor S1647, as well as two newly synthesized derivatives (compounds 12 and 24) that exhibit enhanced selectivity and potency. DHEAS uptake and downstream steroid metabolism were quantified using LC–MS/MS, while DHEAS-induced cell proliferation was analyzed using [³H]thymidine incorporation. SOAT inhibition markedly reduced sodium-dependent DHEAS uptake, resulting in decreased intracellular estradiol synthesis and suppression of estrogen-dependent proliferation without cytotoxicity. These findings confirm that SOAT is a critical upstream regulator of intracrine estrogen biosynthesis in breast cancer cells and highlight compounds 12 and 24 as promising candidates for further preclinical development aimed at reducing local estrogen production.

**Supplementary Information:**

The online version contains supplementary material available at 10.1038/s41598-026-47803-0.

## Introduction

Breast cancer remains the most frequently diagnosed malignancy and the leading cause of cancer-related death among women worldwide^1^. In 2018, an estimated two million new cases were reported globally, with the majority occurring in postmenopausal women, and a substantial proportion resulting in mortality^2^. It is estimated that 70–80% of breast cancers are estrogen receptor (ER)-positive, in which tumor cell proliferation is promoted by estrogens, making the ER pathway a major therapeutic target in breast cancer management^3–5^. In premenopausal women, estrogens are primarily synthesized in the ovaries. After menopause, systemic estrogen levels decline sharply due to the loss of ovarian function. However, peripheral estrogen formation continues and partially compensates for this deficiency, helping to sustain several beneficial estrogen-dependent physiological functions^6^. Local estrogen production in postmenopausal women mainly occurs in peripheral tissues, such as adipose tissue, skin, and mammary glands via the sulfatase and aromatase pathways starting from sulfated steroid precursors such as estrone sulfate (E_1_S) and dehydroepiandrosterone sulfate (DHEAS)^7–11^.

Several studies have demonstrated a strong association between circulating steroid hormones and breast cancer risk. Hankinson et al.^12^ reported that case subjects had significantly higher plasma levels of estrone (E_1_), estradiol (E_2_), E_1_S, and DHEAS compared with controls. DHEAS is particularly noteworthy, because it circulates at concentrations 100,000–200,000 times higher than E_2_^13^, and women with DHEAS levels in the upper three quartiles show a higher risk of developing breast cancer compared with those in the lowest quartile^12,14^. Kalogera et al.^15^ reported that the plasma levels of DHEAS were significantly higher in women with lobular neoplasia compared to those with benign breast disease. Furthermore, DHEAS levels were higher in lobular neoplasia than in invasive ductal carcinoma and showed a significant difference compared to the ductal carcinoma. As reported by Thijssen et al.^16^, estrogen levels are higher in breast tissue than in plasma, particularly in malignant tissue. This difference likely reflects local enzyme activities converting androgens to estrogens, as noted by Berstein et al.^17^ and Vermeulen et al.^18^. These observations suggest that tissue hormone levels may be more directly relevant for assessing breast cancer risk than plasma measurements. Consistent with clinical evidence, elevated DHEAS levels have also been reported in postmenopausal patients with lobular neoplasia. Breast cancer incidence peaks after menopause, despite circulating free estrogens being below biologically active levels^12^, a paradox explained by the persistence of sulfated steroid reservoirs, particularly adrenal-derived DHEAS, which enable local estrogen production within tumor tissues^19^. This process, known as intracrine estrogen synthesis, accounts for nearly all active sex steroid production in postmenopausal women, as these hormones are predominantly synthesized within peripheral target tissues from adrenal precursors such as dehydroepiandrosterone (DHEA) and its sulfate ester DHEAS^20^. Steroid sulfatase (STS), which hydrolyzes steroid sulfates such as E_1_S and DHEAS into their biologically active forms^21,22^, plays a pivotal role in tumor growth, and its inhibition has emerged as a promising therapeutic strategy for ER-positive breast cancer^23,24^. Due to its negative charge, DHEAS requires carrier-mediated uptake, and several transporter families have been identified to facilitate its entry into ER-positive breast cancer cells^25^.

The solute carrier family SLC10 consists of four orphan members (SLC10A3–SLC10A5 and SLC10A7) with unidentified endogenous substrates and three well-characterized transporters: the intestinal apical sodium-dependent bile acid transporter (ASBT, gene symbol *SLC10A2*), the hepatic Na^+^/taurocholate co-transporting polypeptide (NTCP, gene symbol *SLC10A1*), and the sodium-dependent organic anion transporter (SOAT, gene symbol *SLC10A6*)^26–28^. SOAT facilitates the cellular uptake of sulfated steroid hormones in steroid-responsive tissues and organs^29–31^. SOAT is highly expressed in breast cancer tissues, particularly in precancerous and malignant lesions, such as ductal hyperplasia, intraductal carcinoma, and invasive ductal carcinoma^32,33^. Functional studies have demonstrated that SOAT-mediated E_1_S uptake stimulates the proliferation of breast cancer cells in vitro. Conversely, inhibition of SOAT effectively blocked this proliferative effect. Together, these findings highlight SOAT as a promising drug target for novel anticancer strategies^32^.

The phenylsulfonylaminobenzanilide compound S1647 has recently been identified as a potent SOAT inhibitor^34^. In our recent work, we systematically evaluated newly synthesized and commercially available S1647 derivatives for their inhibitory potency and specificity, demonstrating that while S1647 inhibited DHEAS transport in SOAT-transfected human embryonic kidney 293 (HEK293) cells with half-maximal inhibitory concentration (IC₅₀) of 3.5 µM, the derivatives 12 (5-chloro-substituted derivative) and 24 (B-thiophene-substituted derivative) showed stronger effects with IC₅₀ values of 1.9 µM and 0.6 µM, respectively^35^. Based on these findings, our aim was to investigate the effects of these new inhibitors on SOAT-mediated DHEAS-induced proliferation using SOAT-overexpressing MCF-7 cells, a well-established model of intracrine estrogen synthesis after DHEAS incubation^36^.

## Results

To investigate the effects of pharmacological SOAT inhibition on SOAT-mediated DHEAS transport, cell proliferation, and intracrine estradiol synthesis in breast cancer, we generated MCF-7 cells stably overexpressing human SOAT (MCF-7_SOAT) using the Flp-In system. This overexpression model allows us to study the specific role of SOAT under controlled conditions and to evaluate the impact of SOAT inhibitors on key cellular processes involved in hormone-dependent tumor growth.

### SOAT expression and inhibitor selection

**Fig. 1:**
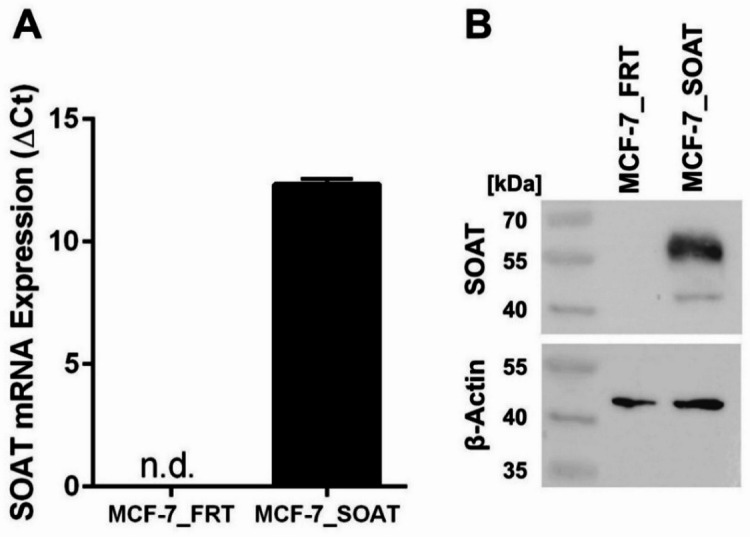
Overexpression of human SOAT in MCF-7 cells. (**A**) SOAT mRNA expression levels were determined by qPCR in MCF-7_FRT control cells and in SOAT-expressing MCF-7_SOAT cells. Data are presented as relative expression normalized to the housekeeping gene β-actin and are shown as mean ± SD of triplicate determinations. (**B**) SOAT protein expression levels were assessed by Western blot analysis. Beta-actin served as the loading control. SOAT was detected using the anti-SOAT antibody (HPA016662, Sigma-Aldrich, 1:500). Beta-actin was detected using the anti-β-actin antibody (A5441, Sigma-Aldrich, 1:5000). Uncropped images can be found in Supplementary Figure S1.

To confirm stable expression of human SOAT in MCF-7 cells, both mRNA and protein levels were analyzed by quantitative real-time PCR (qPCR) and Western blot, respectively. The qPCR analysis revealed no detectable SOAT mRNA expression in MCF-7_FRT cells, whereas MCF-7_SOAT cells exhibited high levels of expression (Fig. 1A). The results of the Western blot analysis consistently confirmed the presence of the SOAT protein in MCF-7_SOAT cells but did not detect it in MCF-7_FRT cells (Fig. 1B and Supplementary Fig. S1).

**Fig. 2:**
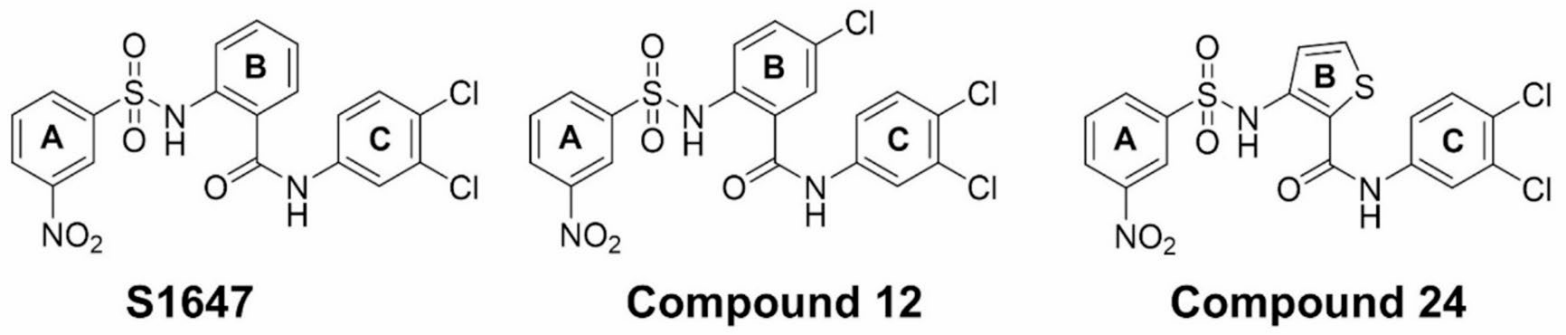
Chemical structures of the SOAT inhibitors, phenylsulfonylaminobenzanilide compound S1647, and the 5-chloro-substituted (compound 12) and B-thiophene-substituted (compound 24) derivatives.

In the present study, the phenylsulfonylaminobenzanilide compound S1647 as well as the 5-chloro-substituted (compound 12) and B-thiophene-substituted (compound 24) derivatives were used as pharmacological SOAT inhibitors (Fig. 2). Lactate dehydrogenase (LDH) release assays were performed to assess potential toxicity of S1647, compound 12, and compound 24 in MCF-7_FRT and MCF-7_SOAT cells following 24-hour treatment at 0.1 and 10 µM. LDH release was quantified as an indicator of membrane integrity. None of the compounds showed any cytotoxicity (Supplementary Fig. S2).

### Functional assessment of SOAT-mediated DHEAS uptake

**Fig. 3:**
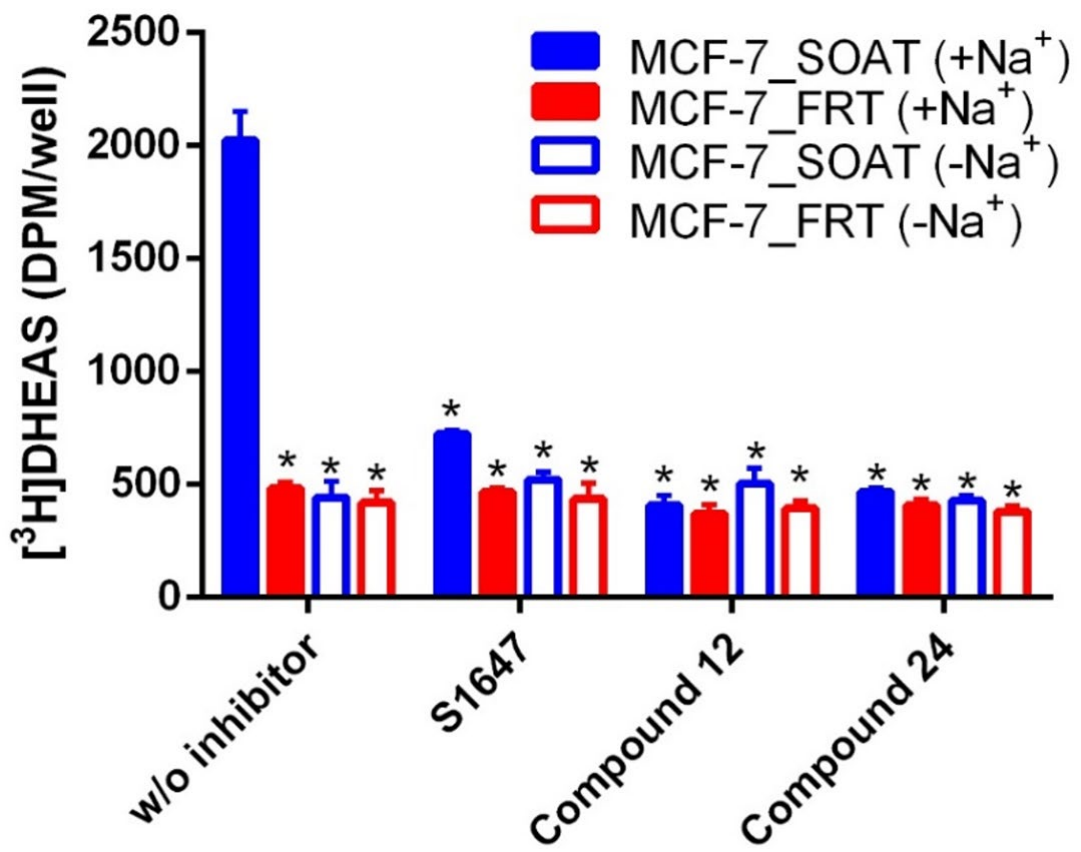
Effect of SOAT inhibitors on [³H]DHEAS uptake. MCF-7_FRT and MCF-7_SOAT cells were preincubated for 5 min with the SOAT inhibitors S1647, compound 12, and compound 24 (each at 10 µM) and subsequently exposed to [³H]DHEAS (1 µM) for a 20 min uptake phase. Uptake assays were performed in transport buffer with sodium (+ Na^+^) and in sodium-free buffer (-Na^+^). Each value represents the mean ± SD of quadruplicate determinations. *Significantly lower DHEAS uptake compared to the positive control (MCF-7_SOAT, +Na^+^, without inhibitor) with *p* < 0.05 according to two-way ANOVA and Tukey’s multiple comparisons test.

To verify functional SOAT carrier expression in the cell membrane of MCF-7_SOAT cells, we performed transport experiments using [³H]DHEAS as the test substrate under both sodium-containing (+ Na^+^) and sodium-free (–Na^+^) conditions. [³H]DHEAS uptake was significantly higher in the MCF-7_SOAT cells compared to MCF-7-FRT cells and was strictly sodium-dependent (Fig. 3). This sodium-dependent [³H]DHEAS uptake in MCF-7_SOAT cells was significantly and efficiently blocked following a 5 min preincubation with S1647, compound 12, or compound 24, each at 10 µM inhibitor concentration, demonstrating strong pharmacological inhibition of SOAT-mediated [³H]DHEAS transport (Fig. 3).

### Effect of SOAT inhibition on MCF-7 cell proliferation

**Fig. 4:**
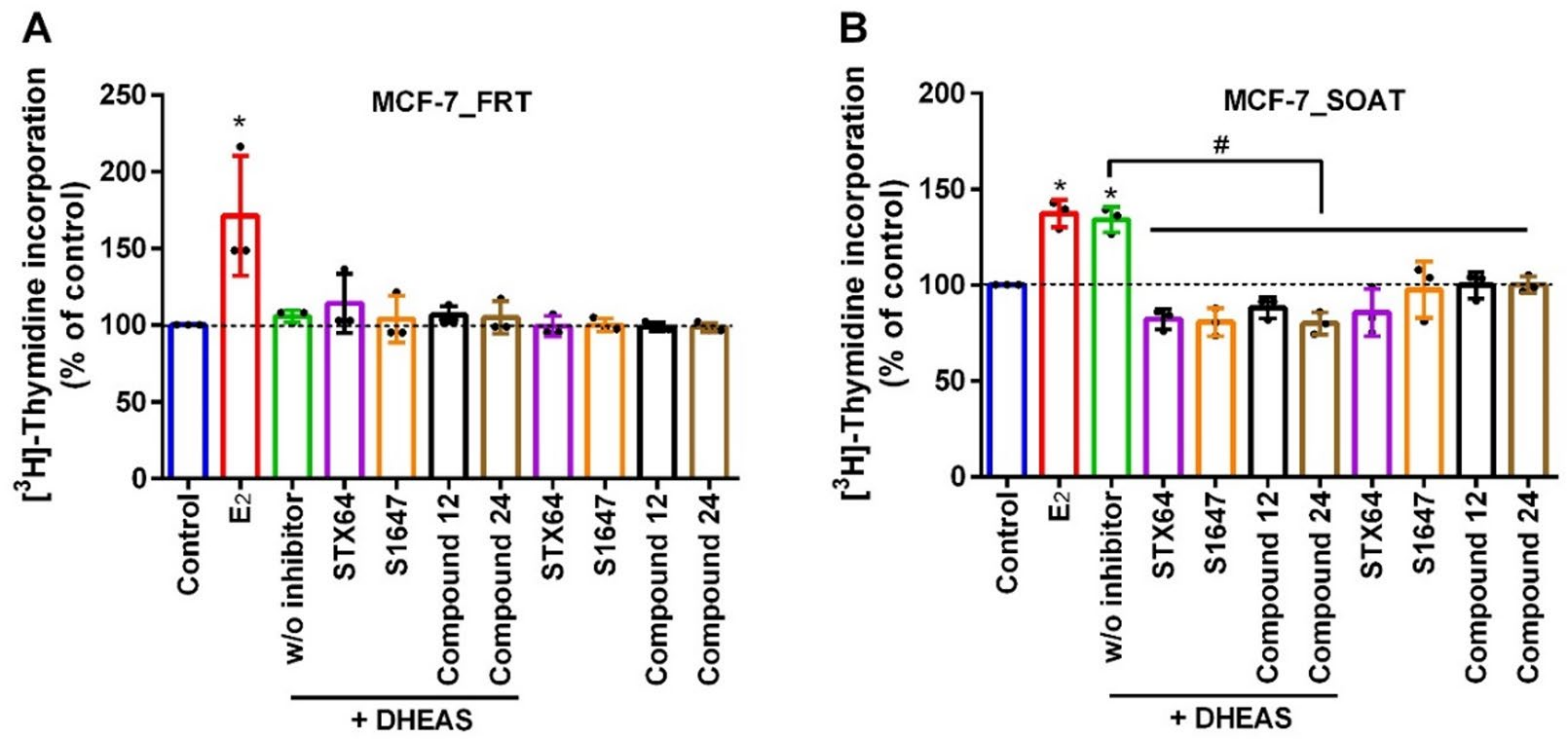
Proliferation of MCF-7 breast cancer cells. Cell proliferation assays were performed in MCF-7_FRT (**A**) and MCF-7_SOAT cells (**B**). Cells were seeded in 48-well plates in standard culture medium for 24 h and then maintained for 3 days in steroid-depleted medium (phenol-red free DMEM supplemented with 10% DCC-FCS) to induce hormone starvation. After starvation, cells were pre-incubated with the indicated SOAT inhibitors (10 µM) for 10 min, followed by stimulation with DHEAS (10 µM) for 3 days. E_2_ (10 nM) and STX64 (1 µM) were included as controls. Proliferation was quantified after 3 days of treatment by incorporation of [³H]thymidine. Data present means ± SD of three independent experiments that were performed in quadruplicate determinations. ^*^Significantly higher proliferation compared to control and ^#^significantly lower proliferation compared to the non-inhibited control w/o inhibitor with *p* < 0.05 according to one-way ANOVA and Tukey’s multiple comparisons test.

Subsequently, the effect of SOAT inhibition on MCF-7 cell proliferation was assessed by means of [³H]thymidine incorporation, given that intracellular DHEAS is metabolized into active estrogens that promote cell proliferation. To eradicate estrogenic hormones derived from fetal calf serum, (FCS), cells were cultivated in charcoal/dextran-stripped FCS, (DCC-FCS) medium in a process of hormone deprivation. As a positive control, treatment with 100 nM E2 significantly increased proliferation in both MCF-7_FRT and MCF-7_SOAT cells in comparison to the control w/o E2 treatment, (Fig. 4). Conversely, DHEAS only significantly stimulated proliferative of MCF-7-SOAT cells, (Fig. 4B) but had no effect on MCF-7_FRT control cells, (Fig. 4A). Interestingly, the stimulatory effect on the proliferation of MCF-7_SOAT cells was comparable for E2 and DHEAS. The STS inhibitor STX64, (at 1 µM) was used as additional control as it prevents DHEAS from cleavage of the sulfate group. As expected, STX64 completely blocked the DHEAS-stimulated proliferation of the MCF-7_SOAT cells. Of note, STX64, S1647, compound 12, and compound 24 alone had no effect on the MCF-7 cell [^3^H]thymidine incorporation in the absence of DHEAS, confirming that the compounds were non-cytotoxic under these conditions. In contrast, the combined incubation of DHEAS with the test inhibitors, S1647, compound 12, and compound 24 completely abolished the DHEAS-stimulated cell proliferation (Fig. 4B).

### SOAT-mediated DHEAS uptake drives estrogen biosynthesis

**Fig. 5:**
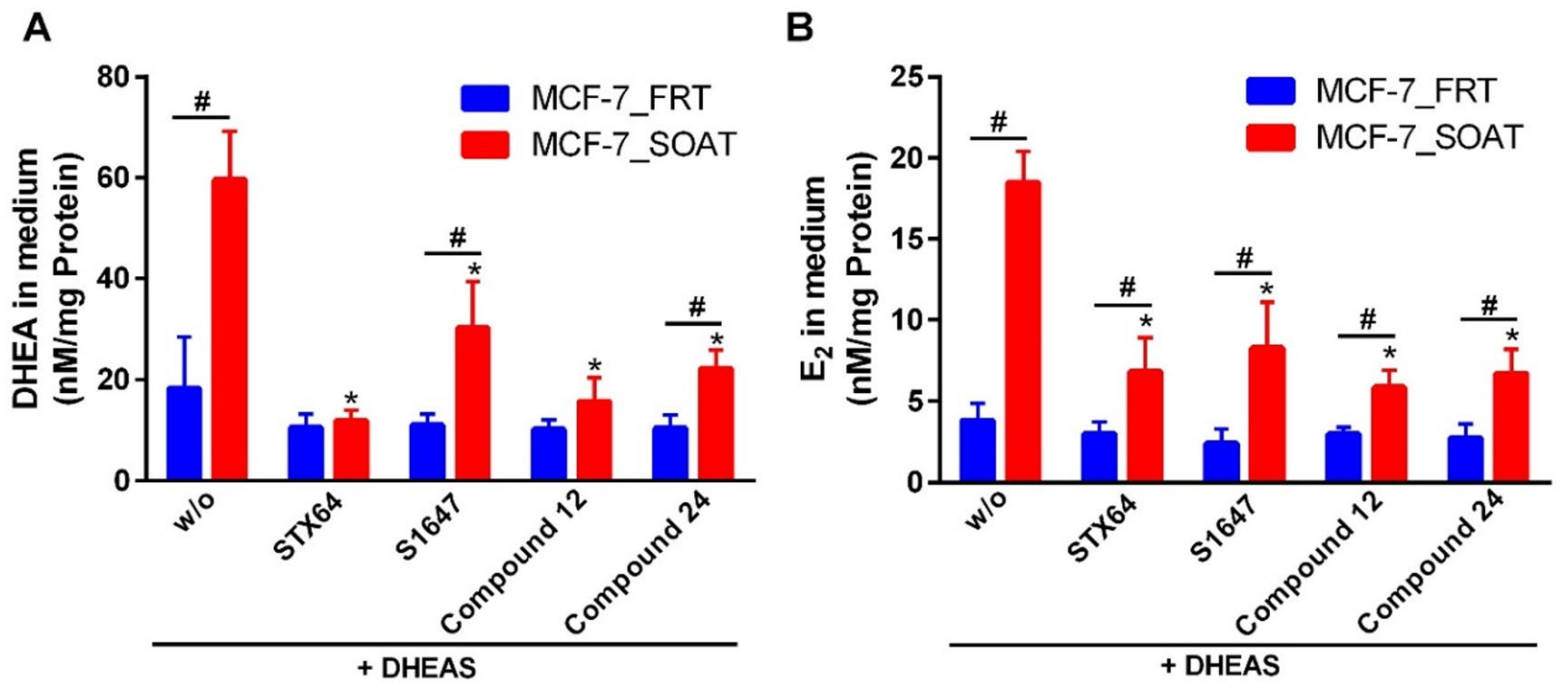
Steroid hormone analysis in cell culture medium. MCF-7_SOAT and MCF-7_FRT cells were pre-incubated for 10 min with SOAT inhibitors (compounds S1647, 12, and 24 at 10 µM) or with the STS inhibitor STX64 at 1 µM and then treated with 10 µM DHEAS for 72 h. The culture media were collected and analyzed by LC-MS/MS to determine the steroid content. DHEA and E_2_ were quantified by LC–MS/MS in positive electrospray ionization mode using compound-specific mass transitions. The concentrations of (**A**) DHEA and (**B**) E_2_ are shown. Data are presented as means ± SD from three independent experiments, each performed in triplicate (total *n* = 9). ^*^Significantly lower compared to control without any inhibitor and ^#^significantly different between the MCF-7_FRT and the MCF-7_SOAT cells with *p* < 0.05 according to two-way ANOVA and Tukey’s multiple comparisons test.

To provide further confirmation that SOAT-mediated DHEAS uptake drives estrogen biosynthesis and proliferation, the levels of steroid hormones were quantified by liquid chromatography-tandem mass spectrometry (LC-MS/MS) in cell culture medium after 72 h of DHEAS stimulation. The results were subsequently normalized to protein content per well (Fig. 5). LC-MS/MS profiling revealed that DHEA and E_2_ were not detectable in the untreated culture media. Only when cells were incubated with DHEAS, levels of DHEA (Fig. 5A) and E_2_ (Fig. 5B) were detectable. Generally, DHEA and E_2_ levels were significantly reduced by all inhibitors (STX64, S1647, compound 12, compound 24). Regarding DHEA formation from DHEAS, STX64 was the most effective inhibitor, followed by compound 12, compound 24, and S1647. S1647 and compound 24 seemed to not fully inhibit SOAT at the used concentration of 10 µM, as there was still a significant difference between the MCF-7_SOAT and MCF-7_FRT cells. Regarding formation of E_2_ from DHEAS, all inhibitors were equally active in reducing the amount of E_2_ formation after DHEAS incubation. However, none of the inhibitors had the potential to fully block E_2_ production to the level of the MCF-7_FRT control cells, indicating that STS and SOAT are not completely blocked at the used inhibitory concentrations.

## Discussion

Estrogen receptor-positive breast cancer represents the most prevalent subtype of breast carcinoma, in which estrogenic signaling plays a central role in tumor growth and progression. In this context, the present study aimed to define the functional role of SOAT-mediated DHEAS transport in facilitating intracrine estrogen formation and promoting breast cancer cell proliferation. Specifically, we characterized for the first time the SOAT inhibitory potential of the phenylsulfonylaminobenzanilide compound S1647 and its newly synthesized derivatives, compounds 12 and 24, on SOAT-mediated DHEAS transport and DHEAS-induced cell proliferation in the ER-positive MCF-7 cell line. Therefore, we evaluated if targeting SOAT-mediated steroid sulfate transport may offer a novel therapeutic strategy for ER-positive breast cancer.

Our previous studies identified the testis as the tissue with the highest SOAT expression. Significant expression levels were also observed in the skin, vagina, kidney, pancreas, placenta, lung, heart and mammary gland^30,37^. More detailed analysis of SOAT expression in the mammary gland by immunochemistry detected the SOAT protein in ductal epithelia of normal breast tissue and found markedly increased expression in ductal hyperplasia, intraductal papilloma, atypical ductal hyperplasia, intraductal carcinoma, and invasive ductal carcinoma^32^. However, there was no clear correlation between SOAT expression and tumor grade, stage, receptor status, or patient age. Previous functional studies of SOAT were primarily performed using SOAT stably transfected HEK293 cells^30,38^. Sorting analyses confirmed the localization of SOAT to the plasma membrane^30,37,39,40^. Systematic transport assays using radiolabeled and unlabeled steroids demonstrated that SOAT specifically mediates the uptake of mono-sulfated steroid hormones including pregnenolone sulfate, androstenediol sulfate, androsterone sulfate, testosterone sulfate, E_1_S, DHEAS, and 16α-OH-DHEAS, with K_m_ values ranging from 11 to 29 µM^30,37,38,41^.

Given SOAT´s critical role in the cellular uptake of sulfated steroids, pharmacological inhibition of SOAT has emerged as a potential strategy to modulate steroid availability in hormone-sensitive tissues such as skin, endometrium, prostate, adipose tissue, and breast^31^. Several bile acids, despite not being SOAT substrates, were identified as potent inhibitors, including taurolithocholic acid sulfate (IC₅₀ = 0.5 µM) and lithocholic acid derivatives^30^. Furthermore, several plant-derived compounds such as betulinic acid and digitonin, as well as synthetic xenobiotic organosulfates (e.g., bromosulfophthalein, 2-sulfooxymethylpyrene/2-SMP, and 4-SMP), demonstrated significant SOAT inhibition^34,42^. Notably, our previous study demonstrated that the SOAT inhibitor 4-SMP completely blocked E_1_S-induced proliferation in T47D breast cancer cells, confirming that SOAT-mediated E_1_S uptake contributes to the proliferation of hormone-dependent breast cancer cells^32^.

Further SOAT inhibition studies have identified a number of small molecules with inhibitory potency in the low micromolar range, including the phenylsulfonylaminobenzanilide compound S1647 (with IC₅₀ of 3.5 µM). Of note, S1647 showed considerable cross-reactivity with the SOAT homologous bile acid transporters ASBT and NTCP^34,35,42^. Recent structure activity relationship analyses demonstrated that halogen substitution patterns significantly affect the potency and target selectivity across these carriers^35^. In particular, the 5-chloro substitution of the B-ring derivative (compound 12) displayed increased selectivity against SOAT (IC₅₀ = 1.9 µM), while substitution of the B-ring with a thiophene moiety (compound 24) markedly enhanced SOAT inhibition potency (IC₅₀ = 0.6 µM). Based on this, the present study used compound 12 and compound 24 to evaluate their impact on SOAT inhibition and inhibition of breast cancer cell proliferation (Fig. 4). Consistent with our previous results, DHEAS-induced proliferation in MCF-7 cells was effectively suppressed by S1647 and its derivatives, compound 12 and compound 24, confirming that pharmacological targeting of SOAT can disrupt the intracrine supply of estrogens in hormone-dependent breast cancer.

**Fig. 6:**
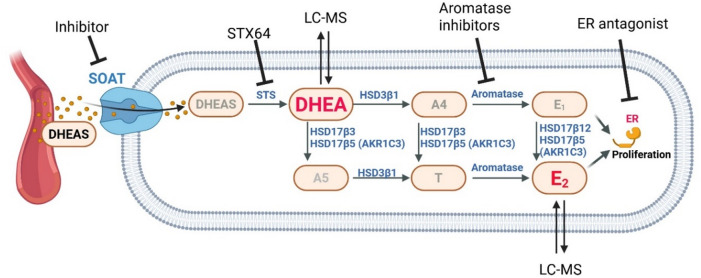
DHEAS metabolic pathway and signaling. Overview of intracrine estrogen synthesis and the target inhibition by SOAT inhibitors (compounds S1647, 12, 24), STS inhibitor STX64, aromatase inhibitors such as anastrozole, and ER antagonists such as tamoxifen. Following the cellular uptake of DHEAS via SOAT, the DHEAS is converted to DHEA by the STS and subsequently converted to active estrogens through sequential metabolism involving 3β-HSD (HSD3β), 17β-HSD (HSD17β), and aromatase. The locally synthesized estrogens activate ER–dependent signaling pathways that finally promote cellular proliferation. Based on their free membrane permeability, DHEAS-derived DHEA and E_2_ were measured in the medium of the cells as surrogate compartment for their intracrine synthesis. Figure created with BioRender.

Consistent with the established model of intracrine steroid synthesis (Fig. 6), DHEAS serves as the predominant circulating adrenal precursor for local estrogen biosynthesis in hormone-dependent tissues. Within breast cancer cells, DHEAS can be sequentially hydrolyzed and converted into active estrogens through the concerted action of steroidogenic enzymes including STS, 3β-hydroxysteroid dehydrogenase (3β-HSD), 17β-hydroxysteroid dehydrogenase (17β-HSD), and aromatase. A recent LC-MS/MS-based study^36^ demonstrated that MCF-7 cells possess full enzymatic capacity to produce DHEA, androstenedione, testosterone, dihydrotestosterone, and E_2_ from DHEAS. Taken together, these findings support the concept that ER-positive breast cancer cells can maintain local estrogenic stimulation even under conditions of systemic estrogen deprivation, particularly in the postmenopausal women. Moreover, the present study revealed that inhibition of SOAT-mediated DHEAS uptake significantly reduced the formation of DHEA and E_2_ in SOAT-transfected MCF-7 cells (Fig. 5). These results establish SOAT as a key upstream regulator of intracrine estrogen biosynthesis by controlling the cellular availability of sulfated steroid precursors.

Hormone receptor-positive (HR-positive) breast cancers account for approximately three-quarters of all breast malignancies, thus making endocrine therapy a vital component of their management^43–45^. However, despite their efficacy, current endocrine therapies are associated with significant side effects and limitations, such as thromboembolic events, endometrial cancer, musculoskeletal pain, bone loss, and sexual or gynecologic adverse effects^46–49^. This highlights the need for alternative strategies to inhibit estrogen-driven tumor growth. In view of these challenges, pharmacological inhibition of SOAT represents a conceptually distinct and promising alternative, as illustrated in Fig. 6. In contrast to the global suppression of estrogen synthesis or receptor blockade, SOAT inhibition acts upstream by preventing the cellular uptake of sulfated steroid precursors such as DHEAS. This process attenuates local estrogen biosynthesis within tumor cells without disturbing systemic endocrine balance. Our data demonstrates that SOAT inhibitors, such as S1647, compounds 12, and compound 24, markedly reduce DHEAS-derived intracrine estrogen formation and estrogen-dependent proliferation in ER-positive MCF-7 cells. Consequently, SOAT emerges as a compelling and druggable target for the development of alternative therapies in hormone-dependent breast cancer.

Beyond SOAT inhibition, it is also important to consider the contribution of other transporter families involved in steroid sulfate uptake. Several carrier systems have been implicated in the cellular uptake of steroid sulfates, including members of the organic anion transporting polypeptide (OATP, gene symbol *SLCO*) and organic anion transporter (OAT, gene symbol *SLC22*) transporter families^50^. Many of these carriers such as OATP1A2, OATP2B1, and OATP3A1 are expressed in breast cancer cells, where they facilitate the uptake of E_1_S and DHEAS, thereby contributing to local estrogen biosynthesis in addition to SOAT. The hepatic bile acid transporter NTCP has also been shown to participate in the uptake of sulfated steroids, however only in hepatocytes^27^. In contrast to the broadly expressed and multi-specific transport systems of the OATP and OAT families, SOAT represents a sodium-dependent active transporter that is highly specific for mono-sulfated steroid hormones. Moreover, SOAT exhibits strong expression in multiple steroid-sensitive peripheral tissues, including the skin, placenta, kidney, pancreas, mammary gland, and testis^30,37,39,41,51^. Importantly, SOAT is co-expressed with STS in breast tissue^32,33,52^, highlighting its coordinated role in the local conversion and regulation of steroid hormones. Collectively, these characteristics establish SOAT as a gatekeeper at the plasma membrane that governs the intracellular availability of sulfated steroid precursors. Its selectivity, sodium-dependent mechanism, and tissue-specific expression pattern make SOAT an attractive and distinctive drug target for modulating intracrine estrogen formation in hormone-dependent breast cancer.

While the present findings provide novel insights into the role of SOAT in intracrine estrogen formation and its pharmacological inhibition, several important limitations should be acknowledged. (I) Transporter selectivity: The target selectivity of S1647, compound 12, and compound 24 toward SOAT, and possible cross-reactivity towards OATP and OAT carriers remains to be comprehensively characterized. (II) In vivo relevance: Although our data demonstrates a clear reduction in DHEAS uptake and estrogen biosynthesis in vitro, in vivo pharmacokinetic and pharmacodynamic studies are required to determine whether these compounds effectively reach tumor tissues and inhibit SOAT-mediated steroid transport under physiological conditions. (III) Metabolic stability and toxicity: The metabolic stability, off-target effects, and potential toxicity of these compounds in vivo have not yet been systematically examined. Such analyses will be essential to define their therapeutic window and evaluate clinical feasibility. (IV) Mechanistic resolution: While SOAT inhibition reduced DHEAS-derived estrogen production, the molecular mechanisms linking SOAT inhibition to downstream changes in steroidogenic enzyme activities (e.g. for STS, 3β-HSD, 17β-HSD, and aromatase) remain to be clarified. Integrated transcriptomic or proteomic approaches could further elucidate these pathways. (V) Tissue-specific expression and regulation: Future studies should investigate how SOAT expression and activity are regulated across different steroid-responsive tissues and under various hormonal or pathological conditions in humans. Understanding this regulatory network will aid in predicting therapeutic responses and minimizing potential side effects.

In conclusion, our study provides compelling evidence that the pharmacological inhibition of SOAT effectively suppresses the uptake of DHEAS and subsequent intracrine estrogen biosynthesis in ER-positive breast cancer cells. By selectively targeting a key upstream transport mechanism, this approach offers a promising and more localized strategy to reduce estrogen-driven tumor proliferation, potentially overcoming the limitations of current systemic endocrine therapies. Further investigation into the specificity, safety, and in vivo efficacy of SOAT inhibitors will be essential to advance this novel concept toward clinical translation.

## Methods

### Materials

All chemicals, unless otherwise stated, were obtained from Sigma-Aldrich (Taufkirchen, Germany). The following unlabeled and deuterium (d)-labeled reference steroids were purchased as indicated from Sigma-Aldrich, Biomol (Hamburg, Germany), or TCI (Eschborn, Germany): DHEAS (Cay15873, Biomol), DHEA (Cay15728, Biomol), E_2_ (Cay10006315, Biomol), and DHEA-d6 (ISO-5170, Biomol). Analytical-grade ultra-pure water (1153332500), methanol (1060352500), and formic acid (5438040100) were from Merck (Darmstadt, Germany). Sep-Pak C18 Plus (360 mg) cartridges (WAT020515) were purchased from Waters Corporation (Milford, MA, USA). The standard compounds were separately dissolved in dimethylsulfoxide (DMSO) at a concentration of 1 mg/mL and stored at −20 °C until further usage. The deuterated standard DHEA-d6 was used as internal standard (IS). Compound numbers of the SOAT inhibitors (compounds 1 = S1647, 12, and 24) refer to previous publication^35^.

### Cell Culture

The human breast cancer cell line MCF-7 gratefully (obtained from Dr. Bernhard Ugele, Department of Gynecology and Obstetrics, University Hospital Munich, Germany) was cultured in a 1:1 mixture of Dulbecco’s Modified Eagle Medium (DMEM) and Ham’s F12 nutrient mixture (Gibco, Carlsbad, CA, USA), supplemented with 10% fetal calf serum (FCS, Gibco), 4 mM L-glutamine (Gibco), 100 U/ml penicillin, and 100 µg/ml streptomycin (Gibco). The cells were maintained at 37 °C in a humidified atmosphere containing 5% CO₂.

### Stably transfected MCF-7 cell lines

To generate MCF-7_FRT cells, parental MCF-7 cells were co-transfected with 1 µg of the pFRT/lacZeo vector (Thermo Fisher Scientific, Waltham, MA, USA) and 1 µg of the pOG44 Flp-recombinase expression vector (Thermo Fisher Scientific), using Lipofectamine 2000 according to the manufacturer’s instructions (Thermo Fisher Scientific). Forty-eight hours after transfection, selection was initiated with 100 µg/ml zeocin. Resistant colonies were then expanded and maintained as the MCF-7_FRT host cell line. To facilitate the stable integration of SOAT, the full-length SOAT coding sequence^30^ was subcloned into the pcDNA5/FRT/TO vector (Thermo Fisher Scientific) using *Hin*dIII and *Xba*I restriction sites. The vector construct was sequence-verified by Sanger sequencing and subsequently introduced into MCF-7_FRT cells with Lipofectamine 2000. Following a 48-hour period, a selective medium containing 100 µg/ml hygromycin B (Carl Roth, Karlsruhe, Germany) was added, and the medium was replaced every three days until resistant colonies were obtained. The resulting cell line (MCF-7_SOAT) was then verified for SOAT expression by quantitative real-time PCR (qPCR).

### Quantitative Real-time PCR

MCF-7_FRT and MCF-7_SOAT cells were seeded in 6-well plates at a density of 1 × 10⁶ cells per well and cultured for 72 h in DMEM/F12 medium. Total RNA was isolated using the Quick-RNA MiniPrep Kit (Zymo Research, Irvine, CA, USA) according to the manufacturer’s protocol. RNA was dissolved in diethylpyrocarbonate-treated water and stored at −80 °C until further use. The RNA concentration and purity were determined spectrophotometrically by measuring absorbance at 260 nm with a NanoDrop spectrophotometer (Thermo Fisher Scientific). First-strand cDNA was synthesized from total RNA using the SuperScript III Reverse Transcriptase Kit (Thermo Fisher Scientific) following the manufacturer’s instructions. Quantitative real-time PCR was performed on a QuantStudio 1 Real-Time PCR System (Applied Biosystems, Darmstadt, Germany) using TaqMan Gene Expression Assays (Applied Biosystems) specific for SOAT (Hs01399354_m1) and β-actin (Hs99999903_m1) as an endogenous control. The PCR reactions were prepared in a total volume of 22 µl containing 4 µl cDNA, 1 µl TaqMan Gene Expression Assay, 10 µl TaqMan Universal PCR Master Mix (Applied Biosystems), and 7 µl nuclease-free water. The thermal cycling conditions were as follows: initial denaturation at 95 °C for 10 min, followed by 40 cycles of 95 °C for 15 s and 60 °C for 60 s. The relative gene expression levels were calculated using the comparative Ct (ΔCt) method, with β-actin as an internal reference.

### Western blot analysis

To extract the proteins, the culture medium was removed, and the cells were washed once with phosphate-buffered saline (PBS, containing 137 mM NaCl, 2.7 mM KCl, 1.5 mM KH_2_PO_4_, 7.3 mM Na_2_HPO_4_, adjusted to pH 7.4). Then, 400 µl of ice-cold RIPA buffer (Sigma-Aldrich), supplemented with a 1:1000 dilution of a protease inhibitor cocktail (Thermo Fisher Scientific), were added to the cells. After an incubation period of 15 min on ice, the cells were lysed by mechanical disruption and incubated on ice for a further 10 min. The lysates were then centrifuged at 13,000 rpm for 15 min at 4 °C and the resulting supernatant collected for protein quantification. Equal amounts of protein (30 µg per lane) were separated by sodium dodecyl sulfate (SDS) polyacrylamide gel electrophoresis on 10% polyacrylamide gels containing 0.3% N, N′-methylene-bis-acrylamide, then transferred onto PVDF membranes (Merck). The membranes were washed with Tris-buffered saline containing 0.05% Tween-20 (TBS-T, containing 137 mM NaCl, 10 mM Tris-HCl, pH 8.0) and then blocked with 5% non-fat dry milk in TBS-T at room temperature with gentle agitation for 1 h. The blocked membranes were then incubated overnight at 4 °C with the following primary antibodies: anti-SOAT (rabbit polyclonal, HPA016662, Sigma-Aldrich; dilution 1:500) and anti-β-actin (mouse monoclonal, A5441, Sigma-Aldrich; dilution 1:5000). After washing with TBS-T, the membranes were incubated for 1 h at room temperature with the respective horseradish peroxidase (HRP)-conjugated secondary antibodies (goat anti-rabbit IgG–peroxidase, A9169, Sigma-Aldrich; 1:5000 and goat anti-mouse IgG–peroxidase, 610–1302, Rockland Immunochemicals, Limerick, PA, USA; 1:5000). Protein bands were visualized using enhanced chemiluminescence and detected using a ChemiDoc imaging system (Bio-Rad, Hercules, CA, USA).

### DHEAS uptake assay

MCF-7_SOAT cells were used for transport experiments with 1 µM [^3^H]DHEAS (88.3 Ci/mmol, NET860250UC, Revvity, Hamburg, Germany). MCF-7_FRT cells were used as a negative control. Cells were seeded onto polylysine-coated 48-well plates, and grown to confluence over 72 h at 37 °C. Then, cells were washed with tempered (37 °C) sodium (+ Na^+^) or choline (-Na^+^) transport buffer (containing 142.9 mM NaCl or 142.9 mM choline chloride, respectively, 4.7 mM KCl, 1.2 mM MgSO_4_, 1.2 mM KH_2_PO_4_, 1.8 mM CaCl_2_, and 20 mM HEPES, adjusted to pH 7.4) and preincubated with transport buffer for 5 min at 37 °C. The medium was replaced by transport buffer containing the respective inhibitor or solvent alone, and cells were further incubated for 10 min at 37 °C. After this preincubation, transport experiments were started by adding transport buffer containing 1 µM of [^3^H]DHEAS. Uptake was terminated by removing the transport buffer and washing with ice-cold PBS, and the plates were kept cool until adding the lysis buffer (1% SDS and 1 N NaOH). Then, the cell-associated radioactivity of [^3^H]DHEAS was quantified by liquid scintillation counting in a TRI-CARB (Packard, Perkin Elmer, San Jose, CA, USA).

### [^3^H]Thymidine incorporation assay

The antiproliferative properties of the newly synthesized inhibitors were investigated through the [^3^H]thymidine incorporation assay. Cells were seeded in 48-well plates in standard culture medium for 24 h and then maintained for 72 h in steroid-depleted medium, representing phenol-red free DMEM supplemented with 10% DCC-FCS, to induce hormone starvation. Then, cells were pre-incubated with the indicated SOAT inhibitors at 10 µM for 10 min, followed by stimulation with DHEAS (10 µM) for 3 days. E_2_ (10 nM) and STX64 (syn. irosustat, Ipsen, Paris, France; M12610001, 1 µM) were included as controls. Proliferation was quantified after 3 days of treatment by incorporation of [³H]thymidine (0.5 µCi per well) during the final 4 h of incubation. Cells were subsequently washed with Tris-HCl buffer and trichloroacetic acid (TCA), lysed with NaOH/SDS, and radioactivity was measured using a scintillation counter TRI-CARB (Packard, Perkin Elmer). The intensity of DNA biosynthesis in cells was expressed in disintegrations per minute (DPM) of radioactive thymidine incorporated in the DNA. The radioactivity observed in untreated control cells was taken as 100%. Values from the tested inhibitors were expressed as a percentage of the control value.

### Determination of steroid levels in cells

Steroid hormone concentrations were analyzed by LC-MS/MS, as previously described^36^. In brief, MCF-7 cells were seeded at a density of 1 × 10⁶ cells/well in six-well plates and maintained in phenol red-free DMEM supplemented with 10% DCC-FCS for 72 h to induce hormone starvation. The cells were first preincubated with SOAT inhibitors at 10 µM for 30 min and then stimulated with DHEAS (10 µM; final DMSO concentration 0.1%). After 72 h, the conditioned media were collected, centrifuged to remove debris and stored at −80 °C until analysis. All samples were spiked with the IS, equilibrated and extracted using solid-phase extraction (Sep-Pak C18 Plus cartridges). After washing and elution with methanol, the extracts were evaporated under nitrogen and reconstituted in a 100 µL water/methanol mixture containing 0.1% formic acid. The reconstituted samples were filtered and transferred to LC vials for mass spectrometric analysis. Steroid profiling in the culture media was performed using a Shimadzu Nexera XS inert UHPLC system (Shimadzu, Kyoto, Japan) coupled to an API 4000 triple quadrupole (AB Sciex, Framingham, MA, USA) for UHPLC multiple-reaction monitoring-mass spectrometry (UHPLC-MRM/MS) analysis. Chromatographic separation was performed using a Hypersil GOLD aQ C18 column (Thermo Fisher Scientific) with a binary gradient of water (0.1% formic acid) and methanol (0.1% formic acid), at a flow rate of 0.5 ml/min. The column temperature was maintained at 50 °C and the autosampler at 4 °C. DHEA and E_2_ were analyzed in positive ion mode using the following mass transitions: DHEA m/z 289.1/271.3 (retention time of 3.91 min) and E_2_ m/z 255.2/159.0 (retention time of 3.42 min). For each sample, 10 µL was injected for UHPLC/MRM-MS analysis.

### Statistical analysis

Statistical analyses were performed using GraphPad Prism 6.07 (GraphPad Software, La Jolla, CA, USA). One-way ANOVA was used to analyze differences between groups, and two-way ANOVA was used to evaluate the results of steroid profiling. Data are presented as the mean ± standard deviation (SD), and *p*-values < 0.05 were considered statistically significant.

## Supplementary Information

Below is the link to the electronic supplementary material.

Supplementary Material 1

## Data Availability

The data generated in this study are available within the article and its supplementary data files or upon request from the corresponding author.
