# Supplementary material for: Novel SOAT inhibitors block DHEAS transport and suppress proliferation in MCF-7 breast cancer cells
Source: Sci Rep. 2026 Apr 10;16:12016. doi: 10.1038/s41598-026-47803-0 (PMC13069112; doi:10.1038/s41598-026-47803-0)
Supplement: Supplementary file 1 — Supplementary Material 1 [file 41598_2026_47803_MOESM1_ESM.pdf]

Novel SOAT Inhibitors Block DHEAS Transport and Suppress Proliferation in MCF-7 Breast Cancer Cells

Emre Karakus[a]*, Silke Leiting[a], Michael Daude[b], Wibke Diederich[b], Joachim Geyer[a]

[a]*Institute of Pharmacology and Toxicology, Faculty of Veterinary Medicine, Biomedical Research Center Seltersberg (BFS), Justus Liebig University of Giessen, Schubertstr. 81, 35392 Giessen, Germany*

[b]*Philipps-Universität Marburg, Fachbereich Pharmazie, Institut für Pharmazeutische Chemie und Zentrum für Tumor und Immunbiologie, Hans-Meerwein-Straße 3, 35043 Marburg, Germany*

*Corresponding author at: Justus Liebig University of Giessen, Institute of Pharmacology and Toxicology, Faculty of Veterinary Medicine, Biomedical Research Center Seltersberg (BFS), Schubertstr. 81, 35392 Giessen, Germany. Phone: +49 641 99 38410

E-Mail: Emre.Karakus@vetmed.uni-giessen.de

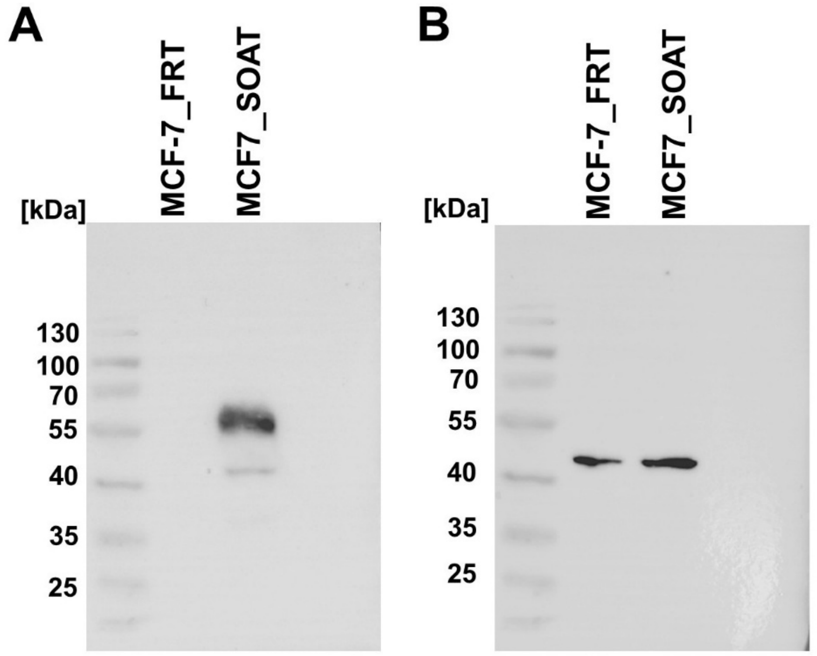

Supplemental Fig. S1. (**A**) SOAT protein expression levels were assessed by Western blot analysis. SOAT was detected using the anti-SOAT antibody (HPA016662, Sigma-Aldrich,

1:500). (**B**) β-Actin was detected using the anti-β-Actin antibody (A5441, Sigma-Aldrich, 1:5000). Full scan of the Western blot shown in **Figure 1B**.

Supplemental Methodology

Cytotoxicity assessment

To confirm that the tested compounds did not induce cytotoxicity during prolonged exposure or at higher concentrations, lactate dehydrogenase (LDH) release was quantified as an indicator of plasma membrane integrity. MCF-7_FRT and MCF-7_SOAT cells were treated for 24 h with S1647 and the derivatives compound 12 and 24 at concentrations ranging from 0.1 to 10 µM. Following incubation, LDH activity in the culture supernatants was determined using a commercially available LDH detection kit (LDH-Cytox Assay, 426401, BioLegend, San Diego, CA). In each well, 100 µL of reaction solution was mixed with 100 µL of culture supernatant in a 96-well microplate. Supernatant from untreated cells served as the negative control, while fresh DMEM was used as a blank. Plates were incubated at 37°C in a humidified atmosphere containing 5% CO_2, and absorbance was measured at 490–620 nm using a microplate reader.

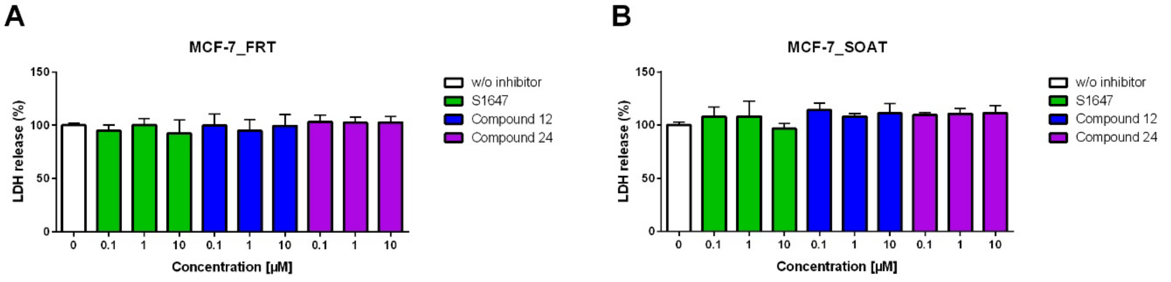

Supplemental Fig. S2. (**A**) Cytotoxicity assessment of SOAT inhibitors by LDH release assay. LDH release in MCF-7_FRT cells following 24 h treatment with the SOAT inhibitors at 0.1, 1, and 10 µM. (**B**) LDH release in MCF-7_SOAT cells under identical treatment conditions. Data represent means ± SD from two independent experiments, each performed in triplicate (n = 6).
